# ABTS On-Line Antioxidant, α-Amylase, α-Glucosidase, Pancreatic Lipase, Acetyl- and Butyrylcholinesterase Inhibition Activity of *Chaenomeles* Fruits Determined by Polyphenols and other Chemical Compounds

**DOI:** 10.3390/antiox9010060

**Published:** 2020-01-09

**Authors:** Igor Piotr Turkiewicz, Aneta Wojdyło, Karolina Tkacz, Paulina Nowicka, Tomasz Golis, Przemysław Bąbelewski

**Affiliations:** 1 Department of Fruit, Vegetable and Plant Nutraceutical Technology, Wrocław University of Environmental and Life Sciences, 37 Chełmońskiego Str., 51-630 Wrocław, Poland; igor.turkiewicz@upwr.edu.pl (I.P.T.); karolina.tkacz@upwr.edu.pl (K.T.); paulina.nowicka@upwr.edu.pl (P.N.); 2 Research Institute of Horticulture, Department of Pomology, Gene Resources and Nurseries, 1/3 Konstytucji 3 Maja Str., 96-100 Skierniewice, Poland; tomasz.golis@inhort.pl; 3 Department of Horticulture, Wrocław University of Environmental and Life Sciences, 24a Grunwaldzki Str., 53-363 Wrocław, Poland; przemyslaw.babelewski@upwr.edu.pl

**Keywords:** Japanese quince, flowering quince, LC-PDA-QTOF-ESI-MS, α-amylase, α-glucosidase, pancreatic lipase, AChE, BuChE, 15-LOX, antioxidant capacity, ABTS on-line, AHC analysis

## Abstract

This study aimed to identify and quantify the chemical composition and polyphenolic profile of 19 cultivars of *Chaenomeles × superba*, *Chaenomeles japonic*a, and *Chaenomeles speciosa* by liquid chromatography coupled with photodiode array detector and quadrupole time-of-flight electrospray ionization mass spectrometry (LC-PDA-QTOF-ESI-MS). Antioxidant (ABTS on-line, ABTS, FRAP, and ORAC), as well as in vitro biological activities, i.e., the ability to inhibit α-amylase, α-glucosidase, pancreatic lipase, acetylcholinesterase (AChE), butyrylcholinesterase (BuChE), and 15-lipoxygenase (15-LOX) were determined. Most of the *Chaenomeles* species and cultivars analyzed in this study have not been examined in this respect until now. Fruits contained 30.26 to 195.05 mg of vitamin C, 0.65 to 1.69 g of pectin, 0.32 to 0.64 g of ash, 0.60 to 3.98 g of sugars, and 41.64 to 110.31 g of organic acids in 100 g fresh weight. The lowest content of total polyphenols showed *C. speciosa* ‘Rubra’ (57.84 g/kg dry weight, dw) while *C.* × *superba* ’Nicoline’ (170.38 g/kg dw) exhibited the highest concentration of those compounds. In the phenolic compounds, polymeric procyanidin fraction predominated (65%) with procyanidin B2, C1, and (−)-epicatechin the most abundant. The antioxidant capacity measured by ABTS assay was mainly formed by polymeric procyanidins and flavan-3-ols, which was confirmed by ABTS on-line profiling. *Chaenomeles* fruits showed high potential for inhibition of α-glucosidase and pancreatic lipase. The analyzed cultivars displayed greater potential for acetylcholinesterase (AChE) inhibition than for butyrylcholinesterase (BuChE). The data indicate that *Chaenomeles* fruits could be regarded as a promising source of bioactive functional food.

## 1. Introduction

*Chaenomeles* species belong to the Rosaceae family (Maloideae subfamily) and have been known widely in China for thousands of years. In Europe, interest in these fruits has been systematically growing over the last twenty years. The generic name is associated with the anatomy of the fruit, it comes from the Greek words “chainein” (divide, open) and “melon” (apple). The systematic affiliation and naming of Maule’s quince were ordered only a dozen years ago and currently four basic species belong to the genus *Chaenomeles*, while in Poland the following three species are mainly grown: *C. speciosa* (Sweet) Nakai (flowering or Chinese quince), *C. japonica* (Thunb.) Lindl. (Japanese quince), and *C. × superba* (Frahm) Rehd. (intermediate quince) which is made from the last two because of the easy crossing within the species [1].

Flowering quince grows up to 2 to 3 meters in height, has spiny shoots, broad and green leaves on the edges, and dark red flowers. The fruits of this species are spherical, slightly elongated, yellow, and aromatic. The Japanese quince is a much lower shrub (1to 1.5 m tall) and more broadly spread. It has smaller, almost round leaves, and the flowers usually have an orange-red color. Yellow fruit with a round shape due to the intense aroma are most often recommended for preserves. Intermediate quince, formed from the aforementioned species, is most commonly found. It is very changeable in appearance, because it has many cultivars differing in height, shape, size of fruit, or the color of flowers (e.g., ‘Crimson and Gold’ blooms in red, and ‘Jet Trail’ in white).

*Chaenomeles* fruit has been widely used in traditional medicine of the Far East, which confirms their presence in the Pharmacopeia of the People’s Republic of China (2010). It describes Fructus *Chaenomeles speciosa*, as a source of medicinal raw materials, but the fruit of other species also have medicinal properties [2]. In vitro and in vivo studies have confirmed the anti-inflammatory, analgesic, antispasmodic, antioxidant, immunoregulatory, and antibacterial effects of this species. The potential to use Japanese quince fruit in the treatment of Parkinson’s disease has also been found [3]. Consumption of the fruit of the *Chaenomeles* genus has been recommended for the following: rheumatism, beri-beri disease, cholera, dysentery, and enterocolitis. In particular, Japanese quince products have been suggested in the therapy for stomach diseases, alleviation of diarrheal symptoms and vomiting, and also protective in liver diseases [1]. Gorlach et al. [4] proved that procyanidin extracted from Japanese quince have proapoptotic activity of HT-29 colon and Caco-2 large intestine carcinoma cells, where fractions containing higher proanthocyanidin oligomers are more active than that of the lower ones.

The aim of this study was to compare the following: (*i*) basic chemical composition (content of dry matter, soluble solid, ash, pectins, L-ascorbic acid, sugars, and organic acids, as well as tritable acidity and pH); (*ii*) the content of bioactive compounds such as polyphenolics including polymeric procyanidins (identification by liquid chromatography coupled with photodiode array detector and quadrupole time-of-flight electrospray ionization mass spectrometry (LC-PDA-QTOF-ESI-MS) and quantification by ultra performance liquid chromatography-photodiode array detector-fluorescence detector (UPLC-PDA-FL); and (*iii*) in vitro biological activities (antioxidant, α-amylase, α-glucosidase, AChE, BuChE, and 15-LOX inhibition activity) in the nineteen cultivars of *Chaenomeles* fruits. Our secondary aim was to determine the relationships between the basic chemical composition, polyphenolic, and specific biological activities of selected species and cultivars of *Chaenomeles* fruits. It should be emphasized that this is the first such comprehensive work characterizing the chemical composition and biological activities including as many as 19 cultivars of three different species of *Chaenomeles*. The research literature contains no reports on the effects of *Chaenomeles* extracts on pancreatic lipase, AChE, BuChE, and 15-lipoxygenase inhibition activity. In addition, research on α-amylase and α-glucosidase activity of specific cultivars, described in this paper, have not been reported by the other authors.

## 2. Materials and Methods

### 2.1. Plant Material and Sample Preparation

Three different species and nineteen cultivars of *Chaenomeles* fruits were used for research. Fruit samples (*C.* × *superba* ‘Crimson and Gold’, ‘Colour Trail’, and ‘Cameo’; *C. japonica* ‘Red Joy’, and *C. speciosa* ‘Nivalis’, and ‘Rubra’) were collected manually from bushes grown in a field trial established in 2016 at the experimental orchard at Wrocław (51°07′ 02.0′′N, 17°04′25.0′′ E). *C.* × *superba* ‘Texas Scarlet’, ‘Nicoline’, ‘Andenken an Karl Ramcke’, ‘Pink Lady’, ‘Flocon Rose’, ‘Hollandia’, and ’Jet Trail’; *C. japonica* ‘Cido’; *C. speciosa* ‘Simonii’; and new genotype (n1) were collected from an experimental field from the Research Institute of Horticulture in Skierniewice (51° 55’ 41.688" N, 20° 9’ 9.896" E). *C.* × *superba* wild and *C. japonica* wild #1 and #2 were collected from wild bushes located near the Centennial Hall in Wroclaw (51° 6′ 26.548′′ N, 17° 4′ 56.782′′ E) in September 2018. Approximately 0.5 kg fruits (of each cultivar) were collected and then were washed with distilled water. The first part of the study included measurements on fresh fruits of dry matter, ash, soluble solids, pH, titratable acidity, pectin, L-ascorbic acid, sugars, and organic acids content.The second part included freeze dried using a freeze drier (Christ Alpha 2–4; Braun Biotech Int., Melsungen, Germany) for 24 h at the pressure of 0.220 mbar. The samples were subsequently ground using a pestle and mortar to a fine powder and stored vacuum packed in a freezer at −80 °C until the analysis but no longer than 5 weeks.

### 2.2. Extraction Procedure

Methanol extracts for determination of polyphenolic compounds were prepared as follows: The freeze-dried powder of fruits (~1 g) was vortexed for 1 min with 6 mL methanol/water/acetic acid/ascorbic acid (30:68:1:1, *v/v/v/m*), sonicated for 20 min (Sonic 6D; Polsonic, Warsaw, Poland) and left for 24 hours at 4 °C. Then, the extract was sonicated again for 20 min, and centrifuged at 19.000 × *g* for 10 min at 4 °C. Finally, the extract was filtred by 0.20 μm hydrophilic PTFE membrane (Millex Simplicity Filter; Merck, Germany) and used for phenolic compounds identification by LC-PDA-QTOF-ESI-MS and quantification by UPLC-PDA. For the determination of antioxidant and in vitro biological activities, the same protocol as that described above was used, but a methanol/water (80:20, *v/v*) with 1% hydrochloric acid mixture was used for extraction.

### 2.3. Physicochemical Analyses

The dry matter was measured using a vacuum dryer (SPT-200; ZEAMiL Horyzont, Kraków, Poland) according to Turkiewiczet al. [5], while the soluble solids content was determined in fresh juices with are refractometer (Atago Rx 5000; Atago Co.Ltd., Kyoto, Japan) and expressed as °Brix. Total ash content was performed as reported previously by Wojdyło et al. [6]. Pectin content was analyzed according to the Morris method described by Pijanowski et al. [7] and expressed as g per 100 g of fresh weight (fw).Titratable acidity was determined by titration aliquots of homogenate of fresh fruits by 0.1 N NaOH to an end point of pH 8.1 using an automatic pH titration system (pH-meter type IQ 150; Warsaw, Poland) and expressed as g of malic acid per 100 g fw. The pH was measured with the same equipment. 

L-ascorbic acid was analyzed according to the HPLC method described previously by Wojdyło et al. [8], and expressed as milligrams per 100 g fw. Sugars were determined by HPLC-ELSD while organic acids by UPLC-PDA method as described previously by Wojdyło et al. [6]. All samples were assayed in triplicate and the results were expressed as g of total sugar content or g of organic acid per kg of fw, respectively.

### 2.4. Identification and Quantification of Phenolic Compounds by the LC-PDA-QTOF-ESI-MS and UPLC-PDA Methods

Identification and quantification of polyphenols from *Chaenomeles* fruits was carried out using an Acquity UPLC system (Waters Corp., Milford, MA, USA) equipped with a photodiode detector (PDA) with binary solvent manager (Waters Corp., Milford, MA, USA) series with a mass detector G2 Q/TOF Micro mass spectrometer (Waters, Manchester, UK) equipped with an electrospray ionization (ESI) source operating in negative modes. An Acquity UPLC BEH C18 column (2.1 × 100 mm, 1.7 µm; Waters Corporation, Milford, USA) at 30 °C was used to perform the chromatographic separation of 5 µL of each sample. Elution at a flow rate of 0.42 mL/min was completed within 15 min using a sequence of elution modes, linear gradients and isocratic. The mobile phase was composed of solvent A (4.5% formic acid) and solvent B (acetonitrile). Samples were eluted according to a linear gradient: 0 to 12 min, 1% to 25% B; 12 to 12.5 min, 100% B; 12.5 to. 13.5 min, 1% B; and, then, held constant to re-equilibrate the column. Analysis was carried out using full scan, data-dependent MS scanning from *m/z* 100 to 1700. Leucine enkephalin was used as the mass reference compound, to ensure that mass was measured accurately, at a concentration of 500 pg/µL. The mass spectrometer was operated in a negative ion mode and set to the base peak intensity (BPI) chromatograms and scaled to 12,400 counts per second (cps) (=100%). The optimized MS conditions were as follows: capillary voltage of 2000 V, cone voltage of 35 V, source and desolvation temperature were of 100 and 250 °C, respectively, and desolvation gas (nitrogen) flow rate of 300 L/h. Collision-induced fragmentation experiments were performed using argon as the collision gas, with voltage ramping cycles from 0.3 to 2 V. The characterization of the single components was carried out via the retention time and the accurate molecular masses. Phenolic acids, flavan-3-ols and flavonols compound were optimized to their estimated molecular masses [M-H]^−^ in the negative mode before and after fragmentation. The data were collected by MassLynx™ 4.1 ChromaLynx Application Manager (Waters Corp., Milford, MA, USA) software.

For quantification, elution was the same gradient as LC-PDA-QTOF-ESI-MS analysis. The PDA spectra were measured over the wavelength range of 200 to 600 nm in steps of 2 nm. The runs were monitored at the following wavelengths: flavan-3-ols at 280 nm, phenolic acids at 320 nm, and flavonols at 360 nm. Retention times (R_t_) and spectra were compared with those of pure standards. Quantification was achieved by injection of solutions of known concentrations ranging from 0.05 to 5 mg/mL (*R*^2^ ≤ 0.9998) made from (−)-epicatechin, (+)-catechin, procyanidin B1, B2, B3, and C1, chlorogenic acid, neochlorogenic acid, 3,5-di-caffeoylquinic acid, quercetin and kaempferol -3-*O*-glucoside, and -3-*O*-rutinoside. 4-*p*-Coumaroylquinic acid was expressed as caffeic acid. Acylated quercetin and kaempferol were expressed as quercetin and kaempferol-3-*O*-glucoside, respectively. All samples were assayed in triplicate and the results were expressed as g per kg of dry weight (dw).

### 2.5. Quantification of Polymeric Procyanidins by the UPLC-PDA-FLMethod

Analysis of polymeric procyanidins was performed by the phloroglucinolysis method as described previously by Wojdyłoet al. [8]. The analysis was carried out on a UPLC system Acquity (Waters Corp., Milford, MA, USA) consisting of a binary solvent manager and fluorescence detector (FL). The fluorescence detection was recorded at an excitation wavelength of 278 nm and an emission wavelength of 360 nm. The calibration curves, which were based on peak area, were established using (+)-catechin, (−)-epicatechin, and procyanidin B1 after phloroglucinol reaction as (+)-catechin- and (−)-epicatechin-phloroglucinol adduct standards. All incubations were done in triplicate. Results were expressed as g per kg of dw.

### 2.6. Determination of Antioxidant and In Vitro Biological Activities

Antioxidant activities were determined using the ABTS method described by Re et al. [9] and the FRAP method described by Benzie and Strain [10]. The ORAC assay was determined following the method previously described by Ou et al. [11]. All samples were assayed in triplicate and the results were expressed as mM of Trolox per 100 g of dw.

The inhibition of α-amylase, α-glucosidase, pancreatic lipase acetylcholinesterase, and butyrylcholinesterase were measured as reported previously by Wojdyło et al. [12] and 15-lipoxygenase inhibition activity was measured using ferric oxidation of xylenol orange (FOX) assay previously described by Chung et al. [13].

All samples were assayed in triplicate and the results were expressed as IC_50_ (mg of dried sample per mL of enzyme) or % of inhibition. All spectrophotometric measurements were performed using a plate reader Synergy H1 (BioTek Instruments, Inc., Winooski, VT, USA). 

### 2.7. Antioxidant On-Line Profiling by HPLC-PDA Coupled with Post-Column Derivatization with ABTS

The antioxidant activity of individual HPLC peaks was measured as reported previously by Turkiewicz et al. [14] using an on-line HPLC antioxidant detector system. The detection wavelength was set at 280 and 734 nm, while the injection volume of sample was 10 μL. The separation was achieved using CADENZA C18 column (75mm × 4.6mm i.d., 3 μm; Tokyo, Japan) with a C18 guard column at 30 °C. The gradient elution solvent was formic acid solution (solvent A, 2%) and acetonitrile (solvent B, 100%) at a flow rate of 0.6 mL/min, 0 to 30 min, 2% to40% B, and up to 45 min column was recognition. ABTS radical cations were produced in accordance with the method described by Re et al. [9]. The second pump delivered the ABTS solution (at a flow rate of 0.2 mL/min) which was mixed with the mobile phase after the first PDA detector. The mixture was guided through PTFE reaction coil (25 m long, 0.25 mm internal diameter, at 40 °C) to a second UV detector, where decolorization of the mixture was detected as a negative peak at 734 nm.

### 2.8. Statistical Analysis

Statistical analysis was conducted using XLSTAT2017, Data Analysis and Statistical Solution for Microsoft Excel (Addinsoft, Paris, France). Significant differences (*p* ≤ 0.05) between means were evaluated by one-way ANOVA and Tukey’s test. Agglomerative hierarchical clustering (AHC) analysis was performed to highlight correlations.

## 3. Results and Discussion

### 3.1. Physiochemical Analysis

Table 1 shows basic chemical composition and physical properties of the analyzed *Chaenomeles* fruits. Dry matter content of the fruits varied significantly (*p* ≤ 0.05) from 10.09% (*C. japonica* n1) to 20.40% (*C*. × *superba* wild). Thomas et al. [15] reported dry matter content of Japanese quince genotypes grown in Sweden and Lithuania as ranging from 10.60% to 11.70%, while Tarko et al. [16] determined dry matter content at the level of 12.90%. Lesińska [17] investigated dry matter in Japanese quince fruit grown in Poland and obtained results from 13% to 18% (average 15.5%) depending on the harvest year. In addition, the key parameter affecting the dry matter content is sun exposure, the shortage of which results in lower dry weight, but also maturity stage, cultivar, climatic conditions, and agrotechnical techniques. Ash content of the analyzed *Chaenomeles* fruits was from 0.32% (*C.* × *superba* ‘Crimson and Gold’) to 0.64% (*C.* × *superba* ‘Pink Lady’) (*p* ≤ 0.05). The results are consistent with those obtained by Rubinskienė et al. [18], which indicates that the range of ash content in Japanese quince fruit is 0.38% to 0.46%. The content of soluble solids (mainly sugars) is an important indicator of the quality of fruit. The sweet taste of fruits depends on the amount of soluble solid content (SSC), which plays an important role for fruit intended for processing, as well as those for direct consumption. The SSC in *Chaenomeles* fruits of selected species and cultivars was from 5.8 (*C. speciosa* ‘Simonii’) to 12.1 °Brix (*C.* × *superba* wild) (*p* ≤ 0.05). Higher values of the SSC were recorded in the fruit of Japanese quince by Rubinskienė et al. [18], i.e., from 14 to 17 °Brix, while results similar to ours (9.4 °Brix) were obtained by Tarko et al. [16] and Ros et al. [19], for 21 different genotypes of *Chaenomeles* fruits SSC ranged from 5.2 to 8.8 °Brix (average 7.1 °Brix). Fruits belonging to the genus *Chaenomeles* are considered rich in pectin compounds, which are located mainly in the fruit pulp. The source of pectin is mainly immature fruit (0.85% to 1.28%), because, during the ripening of fruit, pectin is partially transformed to monosaccharides. The average pectin content in fruits (1.4% of fresh fruit) is equal to or sometimes higher than the values determined in apples [1,15]. The analyzed fruits showed a large variation in pectin content (*p* ≤ 0.05) and the results ranged from 0.65% (*C. japonica* n1) to 1.72% (*C.* × *superba* wild). Undoubtedly, the characteristic feature of *Chaenomeles* fruits is high titratable acidity (TA). For the fruit of *C. japonica*, *C. speciosa*, and *C.* × *superba* values of TA ranged from 3.11 (*C. japonica* ‘Cido’) to 6.16 g malic acid/100 g fresh weight (fw) (*C.* × *superba* wild). Other authors reported acidity values for Japanese quince fruit in the range from 2.6 to 5.6 g of malic acid/100 g fw [19] and 4.10 g of malic acid/100g fw [16]. These values outweigh the acidity of apple juice (0.2 to 0.7 g malic acid/100 g fw) and are comparable with lemon (5.0 to 9.0 g malic acid/100 g fw) [19]. Therefore, the fruits are classified as extremely acidic, unsuitable for direct consumption [19]. The high acidity of the *Chaenomeles* juice was accompanied by a low pH from 2.71 (*C.* × *superba* wild) to 2.99 (*C. speciosa* ‘Simonii’). To confirm the results, Ros et al. [19] determined the pH of *Chaenomeles* fruits genotypes in the range 2.40–2.99 (average 2.60). For comparison, the pH of apple juice is 3.50 to 3.80 and lemon 2.00 to 2.30 [19].

Large variation (*p* ≤ 0.05) was found in the content of L-ascorbic acid. Among the taxa studied, the sample of *C.* × *superba* ‘Hollandia ‘had the highest amount of L-ascorbic acid (195.05 mg/100 g fw) while *C. speciosa* ‘Rubra’ had the lowest amount (30.26 mg/100 g fw). For comparison, Ros et al. [19] determined the average content of L-ascorbic acid in *Chaenomeles* fruit at 128.26 mg/100 g. Bieniasz et al. [20] studied the influence of storage and harvesting year on the content of vitamin C in Japanese quince fruit. They obtained results of L-ascorbic acid content in nine *Chaenomeles* genotypes in the range of 90.0–243.0 and 73.1–172.6 mg/100 g of fresh fruit in two successive years of harvest, respectively. The obtained results are higher than for lemon (50.4 mg/100 g fw), strawberry (60.0 mg/100 g fw), and blackcurrant (86.0 mg/100 g fw) [21].

The *Chaenomeles* fruits are characterized by an extremely low content of sugars in comparison with many other fruits. *C.* × *superba* ‘Texas Scarlet’ was the cultivar with the highest sugar content, 3.98 g/100 g fw (*p* ≤ 0.05), while ‘Jet Trail’ ranked at the other end of the scale (0.44 g/100 g fw). The main identified saccharide was fructose, followed by sorbitol and glucose. Each one accounted for approximately 40.10%, 32.89%, and 26.70% of the total identified sugars, respectively. Xylose was only found in two cultivars of *C.* × *superba*, ‘Crimson and Gold’ and ‘Andenken an Karl Ramcke’, in trace concentrations. For comparison, Hellín et al. [22] analyzed the sugar content in ten *Chaenomeles* genotypes grown in Sweden and Lithuania and found that the main sugars are fructose, glucose, sorbitol, and sucrose. The content of reducing sugars was similar to the values obtained in earlier studies but also the presence of sucrose, maltose, mannitol, stachyose, raffinose, rhamnose, and inositol was reported before [16,22,23,24]. Differences in the quantitative and qualitative composition may result from, among other reasons, the fact that during the analysis of the whole fruit, apart from sugars, the carbohydrate constituents of the structural polysaccharides of the *Chaenomeles* fruit cell wall are also determined, which does not occur during juice analysis. One of the indicators characterizing fruit quality is the ratio of fructose to glucose (fructose is more resistant to heating than glucose, which slows the browning process, e.g., during the manufacture of preserves and marmalades). In the analyzed fruits it was 1.5, while Lesińska [23] found this index for Japanese quince fruit equal to 1.8. In contrast, Tarko et al. [16] reported this ratio equal to 0.3. For comparison, in apples it is 1.8 and in pears 2.0 [23]. 

Contrary to sugars, *Chaenomeles* fruits contain very large amounts of organic acids. In the analyzed samples the following six organic acids were detected: oxalic, maleic, citric, malic, quinic, and shikimic (Table 1). Other typical organic acids normally found in fruits, such as succinic, fumaric, and tartaric acid, were not found in detectable amounts. The content of organic acids in *Chaenomeles* fruits differed between samples (*p* ≤ 0.05). Total content of organic acid ranged from 41.64 (*C. japonica* ‘Cido’) to 110.31 g/kg fw (*C.* × *superba* wild). Malic acid, the main organic acid (81.94% of total acids), was present in the samples at concentrations from 32.08 to 88.75 g/kg of fw, which is consistent with Hellín et al. [22]. The highest amount was found in a sample of *C.* × *superba* wild. The range of quinic acid (15.63% of total acids) was 6.21–16.89 g/kg fw. The remaining acids, i.e., citric, shikimic, oxalic, and maleic were in the minority and accounted for 1.20, 0.89, 0.34, and 0.01%, respectively, of the total amount of organic acids. Another parameter of fruit quality is the ratio of sugars to acids. For fruit intended for direct consumption, it is required that sugars must exceed acids ten-fold. In the case of the analyzed *Chaenomeles* fruits this ratio is only 0.3:1, and thus is even lower than among the fruits of sea buckthorn, wild growing trees, and shrubs or lemons, where this ratio is 1:1. Due to the high acidity, *Chaenomeles* fruits or juices are unsuitable for drinking without the addition of sweeteners. Additionally, for juices a better alternative seems to be the inoculation of the malolactic microorganism *Oenococcus oeni*, which transforms malic acid into lactic acid, characterized by lower acidity. In addition, malic acid is used in the food industry as an acidifying additive, and therefore Chaenomeles juice, like lemon juice, can be used as a natural acidifying agent in a wide range of food products [19,22].

### 3.2. Polyphenol Compounds

**Figure 1:**
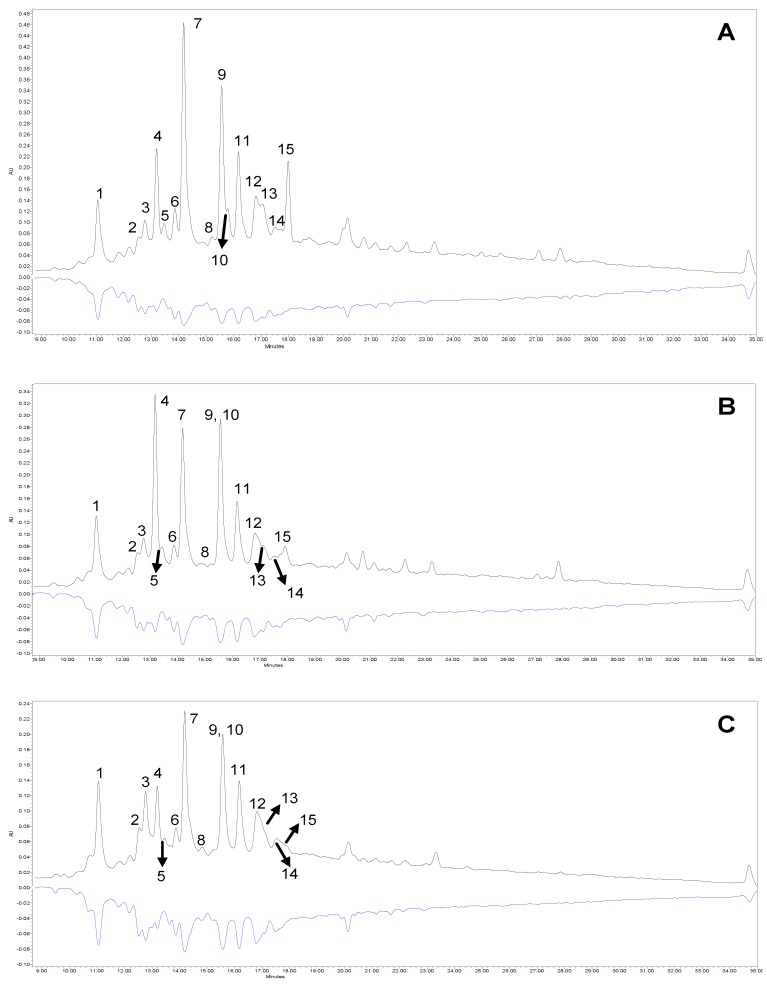
Segment (9.0 to 35.0 min) of chromatographic profiles obtained before and after the derivatization process using the ABTS reagent in samples of *Chaenomeles* × *superba* ‘Texas Scarlet’ (**A**), *Chaenomeles* × *superba* ‘Cameo’ (**B**), and *Chaenomeles speciosa* ‘Nivalis’ (**C**). Peak number identities are displayed in Table 2.

A total of 15 polyphenol compounds were found in the nineteen cultivars of the three *Chaenomeles* species by using LC-PDA-QTOF-ESI-MS (Table 2). Structural formulas of selected phenolic compounds identified in *Chaenomele*s fruits are shown on Figure S1 (Supplementary Material). In the chromatogram profiles (Figure 1) obtained at 280 nm, the labeled peaks 1 to 15followed an elution order. Among these compounds, 13 were flavan-3-ols (monomers and procyanidins dimers, trimers, and tetramers) and two were caffeoylquinic acid derivatives.

The *m/z* values of flavan-3-ol ions were as follows: [M-H]^−^ 289 for monomer of (+)-catechin or (−)-epicatechin, and for B-type procyanidin dimer as [M-H]^−^ 577, trimer as [M-H]^−^ 865, and for tetramer as [M-H]^−^ 1153 [2,25]. Six procyanidin dimers (peaks 1, 7, 8, 9, 14, and 15) were detected at different retention times (R_t_) in the electrospray-ionization quadrupole time-of-flight (ESI-QTOF) mass spectrometry in negative ion mode. All compounds gave the same [M-H]^−^ parental ion at *m/z* 577.13 in accordance with the molecular formula C_30_H_26_O_12_ [26]. Their molecular ions showed a fragment ion (MS/MS) at *m/z* 425.08. In addition, each of these compounds had a fragmentation ion at *m/z* 289.05, which confirms that the procyanidins in *Chaenomeles* fruit are made of (+)-catechin and (−)-epicatechin units. Through analyzing the samples with standards and based on a comparison of R_t_, it was found that peaks 1 and 7 are procyanidin B3 and B2, respectively, which is in accordance with Du et al. [2] and Teleszko and Wojdyło [27]. Moreover, the negative ESI-QTOF spectra of procyanidin B1 and procyanidin B2 gave [M-H-170]^−^ fragment ions at *m/z* 407 from the retro Diels–Alder F reaction of the heterocyclic ring and loss of H_2_O at *m/z* 451 ([M-H-126]^−^) from cleavages betweenC_4_-C_5_ [28]. Therefore, peaks 8, 10, 14 and 15 have been suggested to be procyanidin dimers [25]. Peak **2** (*m/z* 289.06) yielded fragment ions at *m/z* 245.06, 205.03, and 125.01, while peak **9** gave the same fragment ions. Due to the fact that stereoisomers could not be distinguished by mass spectrometry, the retention times have been compared with the standards, and those compounds have been assigned to (+)-catechin and (−)-epicatechin, respectively.

Moreover, according to Bravo et al. [29], the [M-H-44]^−^ fragment at *m/z* 245.06 could result from the loss of a CO_2_ group or the loss of a –CH_2_–CHOH– group and the ion at *m/z* 205.03 is probably due to the loss of a flavonoid A-ring. The presence of an ion at *m/z* 125.01 is considered to be diagnostic for the presence of two (–OH) groups on the A-ring of flavan-3-ols [30]. The elution order of procyanidin monomers, dimers, and trimers was procyanidin B3 < (+)-catechin < procyanidin B2 < (−)-epicatechin < procyanidin C1. Whereas, peaks 3 and 6 with a precursor ion at *m/z* 865.21 and identical molecular formula C_45_H_37_O_18_ were designated as procyanidin trimers. Peak 11 (R_t_ = 16.18 min) exhibited a deprotonated molecule at *m/z* 865.21 and a MS/MS fragment at *m/z* 577.13, 289.06, 245.06, and 125.01. The comparative analysis with standards confirmed that this signal came from procyanidin C1. Additionally, this compound was reported in Japanese quince before by Teleszko and Wojdyło [27] and Lewandowska et al. [31]. Finally, peaks 12 and 13 at Rt = 16.79 and 17.00 min, respectively, with the molecular ion at *m/z* 1153.3 and molecular formula C60H49O24, have been proposed to be procyanidin tetramers. 

With regards to caffeoylquinic acid derivatives, two compounds were detected at Rt = 13.20 min and Rt = 13.33 min. Peaks 4 and 5, with the identical molecular formula C22H27O14, demonstrated the same UV absorption bands and the same [M-H]^−^ at *m/z* 353.08. Moreover, a product ion [M-H-162]^−^ at *m/z* 191.04, which was ascribed to quinic acid, was also the same for both compounds. On the basis of the correct MS and MS/MS data but also the literature [5,26,31], these compounds were designated as 5-O-caffeoylquinic acid (chlorogenic) and 4-O-caffeoylquinic acid (cryptochlorogenic), respectively.

The content of each polyphenol compound was calculated using UPLC-PDA analysis. The flavan-3-ol content including (+)-catechin, (−)-epicatechin, and procyanidin oligomers accounts for 96.02% to 99.85% of all phenolic compounds. This indicates clearly that procyanidins were the main polyphenol compounds in Chaenomeles fruits. Generally, there were three main compounds (procyanidin B2, (−)-epicatechin, and procyanidin C1) in the analyzed species and cultivars of Chaenomeles (Table 3). 

Total phenolic content, calculated as the sum of individual phenolic compounds, varied significantly between genotypes (*p* ≤ 0.05), with *C.* × *superba* ‘Nicoline’ displaying the highest (170.38 g/kg dw), and *C. speciosa* ‘Rubra’ the lowest content (57.84 g/kg dw). Procyanidin B2 was the compound present in the largest amount, in the range from 3.39 (*C. japonica* ‘Red Joy’) to 18.16 g/kg dw (*C.* × *superba* ‘Nicoline’). Analyzing the content of (+)-catechin and (−)-epicatechin in particular species, *C.* × *superba* contained 0.27 to 1.07 and 2.35 to 7.68 g/kg dw, *C. japonica* 0.22 to 0.86 and 1.77 to 4.93 g/kg dw while *C. speciosa* contained 0.69 to 0.99 and 1.97 to 4.99 g/kg dw, respectively. The content of polymeric procyanidins in all the tested genotypes ranged from 34.60 (*C.* × *superba* ‘Color Trail’) to 109.67 g/kg dw (*C.* × *superba* ‘Colour Trail’), with an average of 63.27 g/kg dw. The degree of polymerization (DP) ranged from 2.43 (*C.* × *superba* ‘Colour Trail’) to 4.25 (*C.* × *superba* ‘Pink Lady’), indicating that the analyzed flavan-3-ols were oligomers (2 < DP < 10) with a low degree of polymerization. Moreover, the low DP in the *Chaenomeles* fruit causes them to not be astringent and bitter, such as chokeberry, which also contains significant amounts of procyanidin compounds but with a much higher degree of polymerization [32]. Total phenolic content, as well as polymeric proanthocyanidin concentrations, in this study, were higher than reported by Du et al. [2] and Teleszko and Wojdyło [27]. *C.* × *superba* ’Cameo’ accumulated the greatest amounts of phenolic acids (3.30 g/kg dw), and in ‘Cido’ their content was the lowest (0.15 g/kg dw). Additionally, 4-*O*-caffeoylquinic acid (cryptochlorogenic) was absent in some samples, i.e., *C.* × *superba* ‘Andenken an Karl Ramcke’, ‘Pink Lady’, wild, *C. japonica* wild #2, and *C. speciosa* ’Rubra’.

### 3.3. Antioxidant and In Vitro Biological Activities

The interest in compounds with antioxidant properties has been increasing over the last decades, mainly due to the discovery of the role of active oxygen species in chronic non-infectious diseases, such as cardiovascular diseases and cancer. Currently, there are many methods for determining the antioxidant capacity, tailored to the specifics of the test material and taking into account the potential side reactions. Chemical methods for determining the antioxidant capacity are based on the ability to capture synthetic radicals (ABTS), the reduction of metal ions, for example, iron (FRAP), and the measurement of the antioxidant effect on the rate of oxidation processes occurring in the sample (ORAC).

In this study, these three methods were used after measuring the antioxidant capacity of the test samples (Table 4). The analyzed fruits of selected Chaenomeles species and cultivars showed large variation (*p* ≤ 0.05) among the samples. The highest antioxidant capacity, both ABTS and FRAP, was shown by C. × superba ‘Nicoline’ (20.61 and 21.32 mmol Trolox/100 g dw) while the lowest was shown by C. speciosa ‘Rubra’ (10.91 and 10.24 mmol Trolox/100 g dw). The average antioxidant activity measured by ABTS and FRAP methods from the analyzed species was respectively, for C. × superba (17.39 and 17.18 mmol Trolox/100 g dw), for C. japonica (14.98 and 13.90 mmol Trolox/100 g dw), and for C. speciosa (15.27 and 14.55 mmol Trolox/100 g dw). For comparison, Teleszko and Wojdyło [27] for four Japanese quince cultivars obtained higher values of the activity measured by ABTS and FRAP assays from 44.98 to 68.37 and from 30.73 to 46.57 mmol Trolox/100 g dw. while Du et al. [2] for C. japonica and C. speciosa, determined similar values for ABTS (36.54 and 14.61 mmol Trolox/100 g dw) and for FRAP (11.39 and 2.80 mmol Trolox/100 g dm), respectively. The strongest antioxidant potential measured by the ORAC test was shown by C. × superba ‘Colour Trail’ (66.59 mmol Trolox/100 g dw) and the lowest by C. speciosa ‘Rubra’ and C. japonica wild #2 (33.82 and 33.99 mmol Trolox/100 g dw, respectively). The average ORAC activity for the 19 analyzed varieties of Chaenomeles fruits was 48.35 mmol Trolox/100 g dw and was higher than for the average ORAC value for the artichoke (27.86 mmol Trolox/100 g dw) [5] or grape seeds (36.46 mmol Trolox/100 g dw) [33]. The Global Report on Diabetes WHO [34] states that diabetes had become a serious chronic disease worldwide, and by 2030 could become the seventh greatest killer in the world. The key issue in the fight against type 2 diabetes is finding effective inhibitors of pancreaticα-amylase and intestinal α-glucosidase, responsible for reducing the postprandial glycemia. In addition, agents with α-glucosidase inhibitory are used as oral hypoglycemic agents. Nevertheless, previous studies [35,36] indicate that Chaenomeles fruits may be a potential inhibitor of α-glucosidase. IC50 (mg of dried fruit/mL) for α-amylase and α-glucosidase ranged from 13.88 (C. × superba ‘Nicoline’) to 18.48 (C. speciosa ‘Nivalis’), and from 5.08 (C. × superba ‘Texas Scarlet’) to 15.19 (C. japonica ‘Red Joy’), respectively (*p* ≤ 0.05). Miao et al. [35] analyzed the α-glucosidase inhibition ability of skin from 13 Chaenomeles fruit genotypes in the range 0.05–0.35 mg/mL and flesh 0.04–0.43 mg/mL. To compare, the Actinidia fruits of selected cultivars also showed a higher capacity to inhibit α-amylase (4.13 to 6.40 mg/mL) and α-glucosidase (0.18 to 10.00 mg/mL) [6]. 

The inhibitory activity of pancreatic lipase is used in the prevention of obesity, because it is responsible for hydrolyzing more than half of the consumed triglycerides, to low-molecular compounds and free fatty acids [37]. Therefore, it reduces the amount of fat absorbed into the blood stream and can be used for weight loss control. Among the *Chaenomeles* species and cultivars with reference to the inhibitory activity toward pancreatic lipase, significant differences (*p* ≤ 0.05) were observed. The inhibitory effect (IC_50_) of the analyzed fruits ranged from 0.04 (*C.* × *superba* ‘Andenken an Karl Ramcke’ and *C. speciosa* ‘Rubra’) to 0.35 mg/mL (*C. japonica* wild #1). It should be noted that for five analyzed cultivars, i.e., *C.* × *superba* ‘Color Trail’, ‘Flavon Rose’, ‘Hollandia’, wild, and *C. japonica* wild #2, the values of pancreatic lipase inhibition were designated as <0.01. This means that a lower concentration of *Chaenomeles* had the greatest inhibitory potential. The results were similar to those obtained by Nowicka et al. [37] in 20 peach cultivars in the range from 0.07 to 2.06 mg/mL. It should be emphasized that so far there are no data on the activity of *Chaenomeles* fruit in the literature as regards the inhibition of pancreatic lipase.

Alzheimer’s disease is considered as one of the most prevalent neurodegenerative disorders and accounts for more than 80% of dementia worldwide in the aged population. It is estimated that by 2050, three new case may develop every minute [38]. Acetylcholinesterase (AChE) and butyrylcholinesterase (BuChE, pseudocholinesterase) are key enzymes in the breakdown of an important neurotransmitter, acetylcholine (ACh). Several clinical trials have confirmed that ACh inhibitors could be used to treat this pathology [33,38]. IC_50_ inhibition of AChE and BuChE ranged from 6.65 to 20.42 and from 6.06 to 31.59 mg of dried fruit/mL with significant differences between samples (*p* ≤ 0.05). The cultivars showing the highest ability to inhibit AChE and BuChE were found to be *C. speciosa* ‘Rubra’ and *C. japonica* ’Red Joy’ while the least effective were *C. speciosa* ‘Simonii’ and *C. ×superba* wild #1, respectively. The analyzed *Chaenomeles* genotypes showed similar mean AChE and BuChE inhibition values (IC_50_), 13.24 and 15.32 mg/mL, respectively. It is worth noting that Sancheti et al. [39] during in vivo studies in rats observed a positive effect of the ethyl acetate fraction from *Chaenomeles sinensis*, which caused a strong decrease in AChE activity in diabetic rats.

Lipoxygenases (LOXs) are important enzymes in lipid metabolism that convert the polyunsaturated fatty acids, arachidonic acid (AA), and linoleic acid (LA), to their corresponding metabolites. The inhibitors of 15-LOX have mainly been of interest in the treatment of inflammatory conditions. Recently, multiple studies have provided evidence to elucidate the relationship of 15-LOX-1 and cancer cell growth and development [40]. The results of 15-LOX inhibition clearly showed the great variation of obtained values between tested genotypes (*p* ≤ 0.05). The 15-lipoxygenase inhibition activity was expressed as % inhibition at a sample concentration of 5.77 mg/mL. The highest potential was exhibited by *C.* × *superba* ‘Crimson and Gold’ and ‘Flacon Rose’ (99.81% and 98.15%), while the lowest was shown by *C. speciosa* ‘Rubra’ (14.29%). *C.* × *superba* ‘Pink Lady’, ‘Hollandia’, ‘Jet Trail’ and wild obtained values out of the range (>100.00), which means that the used *Chaenomeles* extracts had very strong inhibitory properties against LOX. The obtained results may be a clue to continue research on cell lines and using a simulated digestive system to verify their biological potential. Moreover, it is advisable to carry out in vivo studies, as there is not enough information in this area.

### 3.4. Antioxidant On-Line Profiling by HPLC-PDA Coupled with Post-Column Derivatization with ABTS

Nowadays, sensitive on-line HPLC-ABTS methods for analyzing free radical scavenging activity have been developed. They combine the liquid chromatography system with additional pumps and detectors allowing the individual active components to be characterized with high sensitivity and evaluation of the antioxidant potential of individual compounds from complex mixtures [41]. Figure 1A–C shows the analysis of the three cultivars of *Chaenomeles* (*C.* × *superba* ‘Texas Scarlet’, *C.* × *superba* ‘Cameo’, and *C. speciosa* ‘Nivalis’). The upper chromatogram (positive, black) shows the response after passing through the first detector at a wavelength of 280 nm, and the lower one (negative, blue) is characterized by the response of the eluted compounds after reaction with the radical cation ABTS after passing through the second detector (λ = 734 nm). The high area of negative peaks on the lower chromatogram is proportional to the activity of individual compounds.

The characteristic elevation of the baseline in the middle part of the upper chromatogram (Figure 1A–C) is caused by the presence of polymeric procyanidins. A mirror reflection of this elevation in the lower chromatogram indicates that these compounds exhibit significant antioxidant activity. Comparing the intensity of the negative response for (–)-epicatechin (peak 9), which is the second highest peak in the order, it can be clearly seen that its antioxidant activity is disproportionate, because this response is negligible. Comparing the activity of (–)-epicatechin and procyanidin C1 (peak 11), whose signal in the upper chromatogram is almost 50% lower, they have a very similar response in the lower chromatogram, and hence similar activity. Numerous in vitro and in vivo studies [1] confirm that polyphenol compounds belonging to the flavan-3-ol group have antioxidant and anti-inflammatory properties. Furthermore, the chemical structure of (–)-epicatechin and its polymers makes them better antioxidants than (+)-catechin and its derivatives, but also the type B procyanidins are better antioxidants than the A type procyanidins, and the degree of polymerization (its increase causes an increase in activity) of the compound is important for their pro-health activity [42]. These results are confirmed by Raudone et al. [43], who also observed greater activity of procyanidin oligomers and polymers than (+)-catechin and (–)-epicatechin. The activity of phenolic acids (peaks 4 and 5) is lower than catechins [44], which can be clearly seen in the blue chromatogram. The signal from chlorogenic acid (peak 4) at 280 nm is significant, whereas its response after reaction with a radical cation is very small. It is caused, among others, by the fact that antioxidant activity increase with the number and position of the –OH groups on the molecule. To summarize, among all identified polyphenolic compounds, procyanidin B3, B2, C1, and (–)-epicatechin were found to be predominant in building antioxidant capacity of *Chaenomeles* fruit, in accordance with Zhang et al. [26] and Raudone et al. [43].

### 3.5. Agglomerative Hierarchical Clustering (AHC)

**Figure 2:**
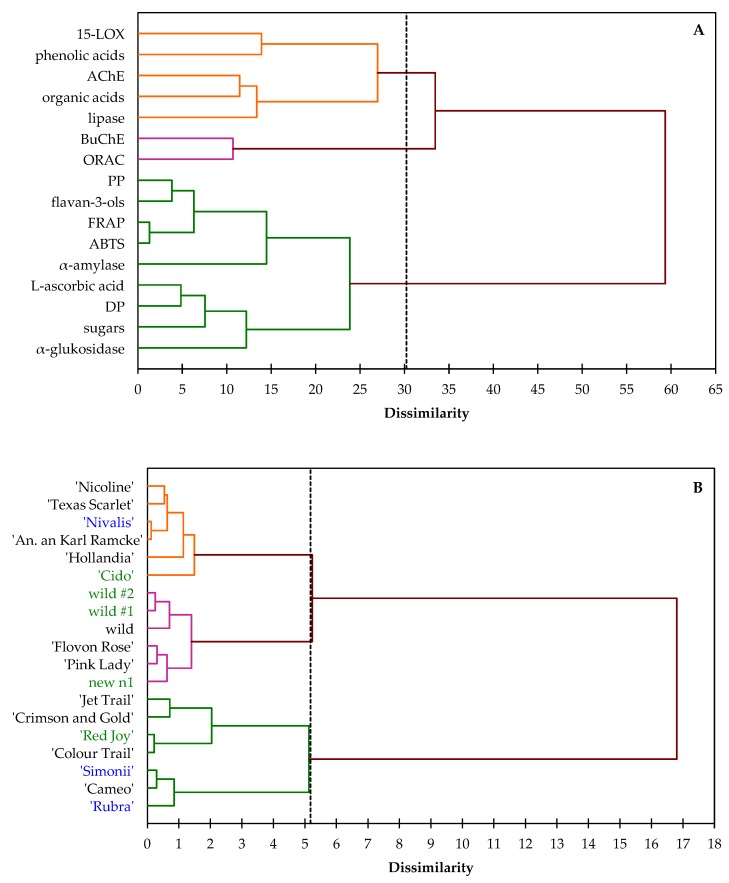
Dendograms of Agglomerative Hierarchical Clustering (AHC) analysis representing dissimilarity relationship of biological activieties and chemical compounds (**A**) and between studied cultivars (**B**) of *Chaenomeles* × *superba* (black), *C. japonica* (green) and *C. speciosa* (blue). PP – polymeric procyanidins; DP – degree of polimerization.

Dendrograms of agglomerative hierarchical clustering (AHC) analysis (Figure 2A,B) showed dissimilarity of biological activities and chemical compounds (A) and between studied cultivars (B) of *Chaenomeles* fruits obtained by Euclidian distance dissimilarity (within the interval 0 to 65 and 0 to 18, respectively) using the aggregation criterion, Ward’s method.

The horizontal axis of the dendrogram represents the dissimilarity between clusters, while the vertical axis represents the objects. Each leaf corresponds to one object and objects that are similar to each other are combined into branches. The greater the height of the horizontal line, the less similar the objects are. By analyzing Figure 2A, it is visible that two groups are approximately the same size, and the third one has only two states. The first group (displayed in orange color) includes objects showing similarity to the second group (displayed in green color). This confirms the calculated Pearson correlation coefficient (*r*), which for phenolic acids and inhibition of 15-LOX is equal to 0.23, and 0.36 for organic acids and inhibition of AChE. In the second group, the branch created between flavan-3-ols and polymeric procyanidins and the activity of ABTS and FRAP is flatter than the others in this cluster. They are more homogeneous with each other (ABTS:flavan-3-ols, *r*=0.86 and ABTS:polymeric procyanidins, *r*=0.76). This is further confirmation (apart from on-line ABTS antioxidant profiling) that flavan-3-ols and their polymers are responsible for the antioxidant capacity of *Chaenomeles* fruits. The third group (displayed in purple) formed between the BuChE and ORAC inhibition activity (*r*=0.41) is more homogeneous with the remaining two clusters (it is flatter on the dendrogram). This is confirmed by comparing the within-class variable, which is almost 70% lower. From the analysis of the dendrogram it can be concluded that inhibition of AChE, BuChE and 15-LOX are influenced by the content of phenolic acids and organic acids, while the polyphenol compounds from the flavan-3-ols group, L-ascorbic acid, and sugars formed the activity of *Chaenomeles* fruit against α-amylase and α-glucosidase. Considering the relationship between the *Chaenomeles* genotypes, in the lower dendrogram (Figure 2B), the three clusters can be seen as three branches that occur at about the same horizontal distance. The two outliers, between ‘Jet Trail’ and ‘Crimson and Gold’, and ‘Red Joy’ and ‘Colour Trail’, fused rather arbitrarily at much greater distances. Moreover, two wild genotypes of *C. japonica* are similar in the context of analyzed parameters and form a cluster with *C.* × *superba* wild with dissimilarity less than one. Not with standing, it can be concluded that there is significant variation within the analyzed species, as well as internal cultivars.

## 4. Conclusions

Physiochemical composition and biological activities of the nineteen *Chaenomeles* species and cultivars evaluated in this study revealed a diverse range of polyphenolic compounds and in vitro biological properties (antioxidant, α-amylase, α-glucosidase, AChE, BuChE, and 15-LOX inhibition activity). The analyzed fruits are rich in polymeric procyanidins and contain a high level of organic acids. *Chaenomeles* × *superba* ‘Nicoline’ displayed the highest total phenol content (170.38 g/kg dw) while *Chaenomeles* × *superba* ‘ColourTrail’ was characterized by the highest concentration of polymeric procyanidins (109.67 g/kg dw). *Chaenomeles* fruits are a good source of malic acid (41.64 to 110.31 g/100 g fw), L-ascorbic acid (30.26 to 1195.05 mg/100 g fw), and pectins (0.65% to1.72%). In addition, *Chaenomeles* × *superba* ‘Nicoline’ showed high potential for inhibition α-amylase and α-glucosidase(as compared with all analyzed species and cultivars), while *Chaenomeles japonica* ‘Red Joy’ proved to be an effective inhibitor for AChE and BuChE. The study established *Chaenomeles* fruits as a source of functional ingredients with possible pharmacological use. However, in order to verify the thesis that *Chaenomeles* fruits are a source of bioactive compounds showing pro-health properties, it is necessary to use in vivo models in further studies. Gastrointestinal systems should be used to determine the bioavailability and digestibility of the *Chaenomeles* bioactive compounds. Therefore, the fruits could be important dietary sources of natural antioxidants.

## Figures and Tables

**Table 1:** Basic chemical composition of selected species and cvs. of *Chaenomeles* fruits.

**Species**	***Chaenomeles* × *Superba***										
**Cultivar**	**Crimson and Gold**	**Texas Scarlet**	**Nicoline**	**Andenken an Karl Ramcke**	**Pink Lady**	**Colour Trail**	**Flocon Rose**	**Hollandia**	**Jet Trail**	**Wild**	**Cameo**
dry matter (%)	13.46 ± 0.20h	16.21 ± 0.21d	17.51 ± 0.30bc	17.13 ± 0.13c	14.76 ± 0.20f	13.82 ± 0.20gh	11.95 ± 0.25ij	15.58 ± 0.18e	13.92 ± 0.22gh	20.40 ± 0.20a	12.31 ± 0.21i
ash content (%)	0.32 ± 0.12j	0.43 ± 0.33fghi	0.48 ± 0.15defg	0.53 ± 0.13bcdef	0.64 ± 0.24a	0.41 ± 0.11ghij	0.46 ± 0.15fg	0.41 ± 0.10ghij	0.51 ± 0.11cdefg	0.57 ± 0.14abcde	0.34 ± 0.44ij
SSC (°Brix)	6.8 ± 0.1h	11.6 ± 0.0b	10.4 ± 0.1c	7.9 ± 0.1f	9.4 ± 0.0d	9.5 ± 0.1d	7.3 ± 0.0g	10.3 ± 0.1c	5.9 ± 0.0j	12.1 ± 0.1a	7.2 ± 0.1g
pectin (%)	1.23 ± 0.10def	1.57 ± 0.10abc	1.62 ± 0.12ab	1.69 ± 0.09ab	1.10 ± 0.10efg	0.99 ± 0.11efgh	0.68 ± 0.18ij	0.98 ± 0.08fghi	1.41 ± 0.10bcd	1.72 ± 0.20a	0.71 ± 0.10hij
TA (g of malic acid/100 g of fw)	4.27 ± 0.13ef	4.60 ± 0.10cde	4.66 ± 0.49cde	5.30 ± 0.15b	4.64 ± 0.49cde	5.20 ± 0.12bc	4.20 ± 0.10ef	4.25 ± 0.15ef	3.45 ± 0.10gh	6.16 ± 0.16a	4.66 ± 0.10cde
pH	2.927 ± 0.01d	2.801 ± 0.00h	2.782 ± 0.01hi	2.738 ± 0.01j	2.772 ± 0.00i	2.897 ± 0.01e	2.855 ± 0.02g	2.842 ± 0.00g	2.975 ± 0.01ab	2.713 ± 0.01j	2.892 ± 0.01ef
L-ascorbic acid (mg/100 g of fw)	40.83 ± 0.55jk	175.32 ± 0.68ab	134.38 ± 0.23cd	144.17 ± 0.50c	111.81 ± 0.88def	47.86 ± 0.74ijk	110.99 ± 0.29ef	195.05 ± 0.30a	70.96 ± 0.55gh	143.09 ± 1.00c	70.29 ± 0.67ghi
Sugars (g/100g fw)											
xylose	0.03 ± 0.00b	nd	nd	0.05 ± 0.00a	nd	nd	nd	nd	nd	nd	nd
fructose	0.58 ± 0.02g	1.80 ± 0.35a	0.60 ± 0.02g	0.51 ± 0.03gh	1.34 ± 0.13bcd	1.10 ± 0.07def	0.94 ± 0.02f	1.62 ± 0.03ab	0.12 ± 0.02i	1.31 ± 0.07cde	1.18 ± 0.04def
sorbitol	0.30 ± 0.00jk	0.95 ± 0.16b	0.56 ± 0.00fgh	0.75 ± 0.03cde	0.72 ± 0.06def	0.72 ± 0.04def	0.47 ± 0.01hij	0.79 ± 0.01bcde	0.22 ± 0.01k	1.40 ± 0.05a	0.37 ± 0.00ijk
glucose	0.29 ± 0.00ghi	1.23 ± 0.26a	0.44 ± 0.02efg	0.29 ± 0.01ghi	0.91 ± 0.07bc	0.88 ± 0.06bc	0.51 ± 0.01def	0.91 ± 0.02bc	0.10 ± 0.01i	0.85 ± 0.04c	0.93 ± 0.03bc
total	1.20 ± 0.02ij	3.98 ± 0.37a	1.60 ± 0.04hi	1.60 ± 0.27hi	2.97 ± 0.26cde	2.70 ± 0.17def	1.92 ± 0.04gh	3.32 ± 0.06bcd	0.44 ± 0.04k	3.56 ± 0.17abc	2.48 ± 0.08fg
fructose:glucose ratio	2.0	1.5	1.4	1.8	1.5	1.2	1.8	1.8	1.2	1.5	1.3
Organic Acids (g/kg fw)											
oxalic	0.06 ± 0.00e	0.70 ± 0.05b	0.24 ± 0.06bcde	0.27 ± 0.00bcde	0.18 ± 0.02bcde	0.23 ± 0.02bcde	0.20 ± 0.03bcde	0.59 ± 0.09a	0.23 ± 0.03bcde	0.28 ± 0.04bcd	0.17 ± 0.02bcde
maleic	0.01 ± 0.00a	0.01 ± 0.00a	0.01 ± 0.00a	0.01 ± 0.00a	0.01 ± 0.00a	0.01 ± 0.00a	0.01 ± 0.00a	0.01 ± 0.00a	nd	0.01 ± 0.00a	0.01 ± 0.00a
citric	0.78 ± 0.07de	0.90 ± 0.12de	0.41 ± 0.02fg	0.75 ± 0.05de	0.66 ± 0.09ef	1.20 ± 0.02ab	1.29 ± 0.10ab	1.35 ± 0.09ab	0.31 ± 0.02g	1.42 ± 0.07a	0.91 ± 0.02cde
malic	62.52 ± 1.33efg	56.12 ± 1.00gh	48.61 ± 1.97hi	64.34 ± 3.54ef	64.87 ± 3.43ef	81.44 ± 2.12abc	62.14 ± 1.70efg	57.64 ± 0.39fg	38.83 ± 1.47j	88.75 ± 1.32a	65.94 ± 2.52e
quinic	10.51 ± 0.16fg	7.52 ± 0.12ij	14.50 ± 0.32bc	12.37 ± 0.15de	9.91 ± 0.04fgh	10.70 ± 0.48efg	14.52 ± 0.18bc	17.28 ± 1.39a	11.50 ± 0.79def	17.15 ± 0.29a	8.70 ± 0.04hi
shikimic	0.11 ± 0.01fg	0.14 ± 0.01fg	0.73 ± 0.05de	0.91 ± 0.07cd	0.12 ± 0.01fg	0.07 ± 0.00g	0.22 ± 0.01efg	0.17 ± 0.00efg	0.65 ± 0.00def	2.70 ± 0.06a	0.10 ± 0.00fg
total	73.99 ± 1.57de	65.07 ± 1.29ef	64.50 ± 2.37ef	78.65 ± 3.82d	75.75 ± 3.55d	93.65 ± 2.64c	78.39 ± 1.96d	77.04 ± 1.96d	51.52 ± 2.26g	110.31 ± 1.12a	75.82 ± 2.60d
sugars:acids ratio	0.2	0.6	0.2	0.2	0.4	0.3	0.2	0.4	0.1	0.3	0.3
Species	***Chaenomeles Japonica***						***Chaenomeles Speciosa***				
Cultivar	**Cido**	**Red Joy**	**Wild #1**	**Wild #2**	**n1 (New)**		**Nivalis**		**Rubra**		**Simonii**
dry matter (%)	13.45 ± 0.15h	10.95 ± 0.15k	17.76 ± 0.14b	14.35 ± 0.15fg	10.09 ± 0.11l		17.04 ± 0.24c		11.95 ± 0.15ij		11.71 ± 0.20j
ash content (%)	0.47 ± 0.23efg	0.35 ± 0.15hij	0.61 ± 0.31abc	0.58 ± 0.22abcd	0.41 ± 0.21ghij		0.43 ± 0.33fghi		0.45 ± 0.15fgh		0.63 ± 0.23ab
SSC (°Brix)	8.4 ± 0.0e	6.3 ± 0.1i	10.3 ± 0.0c	8.6 ± 0.0e	6.3 ± 0.1i		10.5 ± 0.0c		6.6 ± 0.0h		5.8 ± 0.1j
pectin (%)	0.76 ± 0.16hij	0.95 ± 0.15fghij	1.08 ± 0.18efg	0.90 ± 0.10ghij	0.65 ± 0.15j		1.29 ± 0.09cde		0.85 ± 0.15ghij		0.88 ± 0.18ghij
TA (g of malic acid/100 g of fw)	3.11 ± 0.11h	4.90 ± 0.10bcd	5.50 ± 0.15b	5.32 ± 0.12b	3.97 ± 0.10fg		5.44 ± 0.14b		4.58 ± 0.12de		3.45 ± 0.10gh
pH	2.867 ± 0.01fg	2.941 ± 0.01cd	2.965 ± 0.01bc	2.966 ± 0.01bc	2.843 ± 0.00g		2.862 ± 0.00g		2.992 ± 0.01ab		2.994 ± 0.00a
L-ascorbic acid (mg/100 g of fw)	132.33 ± 0.35cde	57.82 ± 0.23hij	101.72 ± 0.21f	114.13 ± 0.21def	91.19 ± 0.57fg		154.97 ± 0.33bc		30.26 ± 0.20k		53.6 ± 0.09hij
	Sugars (g/100 g fw)										
xylose	nd	nd	nd	nd	nd		nd		nd		nd
fructose	1.57 ± 0.01abc	0.18 ± 0.02i	1.11 ± 0.04def	1.02 ± 0.01ef	0.62 ± 0.03g		1.70 ± 0.02a		0.24 ± 0.01hi		0.25 ± 0.02hi
sorbitol	0.66 ± 0.01efg	0.25 ± 0.02k	1.38 ± 0.13a	0.92 ± 0.12bc	0.33 ± 0.02jk		0.88 ± 0.00bcd		0.53 ± 0.03ghi		0.25 ± 0.02k
glucose	1.07 ± 0.03ab	0.17 ± 0.01i	0.65 ± 0.02d	0.62 ± 0.02de	0.42 ± 0.01fgh		1.21 ± 0.01a		0.22 ± 0.00hi		0.15 ± 0.01i
total	3.30 ± 0.05bcd	0.60 ± 0.04jk	3.14 ± 0.20cde	2.56 ± 0.10ef	1.37 ± 0.07hi		3.79 ± 0.03ab		0.99 ± 0.04ijk		0.65 ± 0.04jk
fructose:glucose ratio	1.5	1.1	1.7	1.6	1.5		1.4		1.1		1.6
	Organic Acids (g/kg fw)										
oxalic	0.13 ± 0.02cde	0.20 ± 0.00bcde	0.17 ± 0.05bcde	0.25 ± 0.01bcde	0.65 ± 0.25a		0.26 ± 0.00bcde		0.09 ± 0.01de		0.32 ± 0.08bc
maleic	nd	0.01 ± 0.00a	0.01 ± 0.00a	0.01 ± 0.00a	0.01 ± 0.00a		nd		0.01 ± 0.00a		0.01 ± 0.00a
citric	0.30 ± 0.06g	1.23 ± 0.10ab	1.35 ± 0.04ab	1.33 ± 0.05ab	0.94 ± 0.17cd		1.16 ± 0.09bc		0.81 ± 0.07de		0.75 ± 0.11de
malic	32.08 ± 6.19j	79.51 ± 3.82bc	86.14 ± 1.30ab	86.04 ± 0.78ab	56.20 ± 1.79gh		74.30 ± 3.99cd		67.02 ± 1.20de		47.93 ± 1.74i
quinic	9.05 ± 0.76ghi	9.15 ± 0.36ghi	14.22 ± 0.80bc	15.10 ± 0.56b	13.09 ± 0.20cd		16.89 ± 1.06a		6.21 ± 0.02j		12.33 ± 0.43de
shikimic	0.08 ± 0.01g	1.12 ± 0.08bcd	1.34 ± 0.56bc	0.72 ± 0.53de	0.12 ± 0.01fg		1.48 ± 0.12b		1.17 ± 0.05bcd		0.10 ± 0.01fg
total	41.64 ± 7.04h	91.21 ± 4.36c	103.23 ± 2.58ab	103.47 ± 1.81ab	71.01 ± 1.52de		94.09 ± 5.26bc		75.30 ± 1.30d		61.45 ± 2.37f
sugars:acids ratio	0.8	0.1	0.3	0.2	0.2		0.4		0.1		0.1

nd, not detected; SSC, soluble solid content; TA, tritable acidity; fw, fresh weight. ± Standard deviation, value in the same columns followed by different letters are significantly different at *p* ≤ 0.05 according to Tukey’s test.

**Table 2:** Identification of phenolic compounds in *Chaenomeles* fruits on example *Chaenomeles* × *superba* ‘Texas Scarlet’ by using liquid chromatography coupled with photodiode array detector and quadrupole time-of-flight electrospray ionization mass spectrometry (LC-PDA-QTOF-ESI-MS).

Peak	Compound	R_t_ (min)	λ_max_ (nm)	Molecular Formula	MS [M-H]^−^ (*m/z*)	MS/MS (*m/z*)
**1**	Procyanidin B3	11.09	280	C_30_H_26_O_12_	577.13	425.08/451.00/407.05/289.05
**2**	(+)-Catechin	12.53	240/280	C_15_H_13_O_6_	289.06	245.06/205.03/125.01
**3**	Procyanidin trimer	12.79	280	C45H37O18	865.21	577.13/425.08/289.06
**4**	5-*O*-Caffeoylquinic acid (chlorogenic)	13.20	246/326	C_22_H_27_O_14_	353.08	191.04
**5**	4-*O*-Caffeoylquinic acid (cryptochlorogenic)	13.33	246/326	C_22_H_27_O_14_	353.08	191.04
**6**	Procyanidin trimer	13.84	280	C45H37O18	865.21	577.13/425.08/289.06
**7**	Procyanidin B2	14.19	280	C_30_H_26_O_12_	577.13	425.08/451.00/407.05/289.05
**8**	Procyanidin dimer	15.21	280	C_30_H_26_O_12_	577.13	425.08/289.05
**9**	(−)-Epicatechin	15.58	240/280	C_15_H_13_O_6_	289.06	245.06/205.03/125.01
**10**	Procyanidin dimer	15.80	280	C_30_H_26_O_12_	577.13	425.08/289.05
**11**	Procyanidin C1	16.18	280	C45H37O18	865.21	577.13/289.06/245.06/125.01
**12**	Procyanidin tetramer	16.79	280	C60H49O24	1153.3	865.21/576.12/289.05
**13**	Procyanidin tetramer	17.00	280	C60H49O24	1153.3	865.21/576.12/289.05
**14**	Procyanidin dimer	17.20	280	C_30_H_26_O_12_	577.13	425.08/289.05
**15**	Procyanidin dimer	17.92	280	C_30_H_26_O_12_	577.13	425.08/289.05

**Table 3:** Content of phenolic compounds (g/kg dw) invarious species and cvs. of *Chaenomeles* fruits.

	***Chaenomeles* × *Superba***																	
**Peak no**	**Crimson and Gold**	**Texas Scarlet**		**Nicoline**		**Andenken an Karl Ramcke**		**Pink Lady**		**Colour Trail**	**Flocon Rose**		**Hollandia**	**Jet Trail**			**Wild**	**Cameo**
Phenolic acids																		
4	0.43 ± 0.15ef	1.68 ± 0.20bc		1.34 ± 0.21cd		1.35 ± 0.10cd		0.79 ± 0.13de		0.23 ± 0.08ef	1.65 ± 0.27bc		1.08 ± 0.11cd	1.12 ± 0.19cd		3.04 ± 0.21a		3.05 ± 0.15a
5	0.46 ± 0.12bc	0.35 ± 0.10bc		0.04 ± 0.00c		nd		nd		0.36 ± 0.08bc	0.41 ± 0.22bc		0.45 ± 0.11bc	0.06 ± 0.00c		nd		0.25 ± 0.14bc
Sum	0.89 ± 0.15ef	2.03 ± 0.20bc		1.38 ± 0.00de		1.35 ± 0.31de		0.79 ± 0.20ef		0.59 ± 0.10fg	2.06 ± 0.33bc		1.53 ± 0.18cd	1.18 ± 0.27def		3.04 ± 0.11a		3.30 ± 0.19a
Flavan-3-ols																		
1	0.28 ± 0.08i	2.86 ± 0.22a		1.85 ± 0.21cde		1.47 ± 0.18efg		0.65 ± 0.09hi		1.00 ± 0.12gh	2.02 ± 0.44bcde		1.66 ± 0.36def	1.76 ± 0.15cde		1.75 ± 0.27cde		2.75 ± 0.22a
2	0.27 ± 0.10de	0.29 ± 0.11cde		0.42 ± 0.08bcde		0.37 ± 0.10cde		0.42 ± 0.15bcde		0.75 ± 0.15abcde	0.62 ± 0.22abcde		0.69 ± 0.19abcde	1.07 ± 0.08a		0.86 ± 0.31abcd		0.86 ± 0.28abcd
3	nd	2.86 ± 0.44a		2.64 ± 0.39a		2.40 ± 0.30ab		nd		0.40 ± 0.18f	1.07 ± 0.19de		0.88 ± 0.24def	0.94 ± 0.09def		1.16 ± 0.12de		1.97 ± 0.33bc
6	1.59 ± 0.18c	2.33 ± 0.28b		3.00 ± 0.30a		2.28 ± .21b		1.03 ± 0.18cde		0.87 ± 0.22de	0.91 ± 0.10de		1.48 ± 0.19cd	2.73 ± 0.28ab		1.46 ± 0.17cd		1.39 ± 0.19cde
7	10.92 ± 1.21d	13.40 ± 1.30c		18.16 ± 1.02a		14.19 ± 1.00b		5.53 ± 0.50i		7.90 ± 0.67f	5.74 ± 0.78hi		9.29 ± 0.62e	14.72 ± 1.05b		9.60 ± 0.77e		8.24 ± 0.46f
8	1.27 ± 0.62cde	2.59 ± 0.80a		2.55 ± 0.55a		1.84 ± 0.42bc		1.34 ± 0.42cde		1.28 ± 0.33cde	1.20 ± 0.27de		1.34 ± 0.19cde	2.16 ± 0.55ab		1.17 ± 0.31def		1.42 ± 0.41cd
9	6.8 ± 0.99bc	5.03 ± 0.55d		6.99 ± 0.89b		7.68 ± 0.77a		2.35 ± 0.60gh		5.02 ± 0.63d	2.79 ± 0.49g		4.35 ± 0.55f	6.30 ± 0.68c		4.59 ± 0.70def		4.93 ± 0.66def
10	nd	0.91 ± 0.10a		0.48 ± 0.25abc		nd		0.23 ± 0.11c		nd	0.51 ± 0.21abc		0.65 ± 0.10abc	0.65 ± 0.10abc		nd		0.79 ± 0.13abc
11	3.90 ± 0.54de	5.88 ± 0.52c		7.58 ± 1.12a		6.71 ± 0.66b		1.87 ± 0.28ij		3.05 ± 0.65fgh	3.16 ± 0.33fg		4.12 ± 0.33de	5.84 ± 0.74c		4.31 ± 0.56gh		4.20 ± 0.60de
12	2.39 ± 0.10fgh	3.78 ± 0.25bc		4.36 ± 0.85ab		3.61 ± 0.33cd		1.29 ± 0.00jk		1.81 ± 0.31hij	2.26 ± 0.55gh		2.41 ± 0.33fgh	3.07 ± 0.59de		2.05 ± 0.27hi		2.05 ± 0.55ghi
13	4.28 ± 0.87d	6.03 ± 0.54bc		7.12 ± 0.42a		5.81 ± 0.77c		2.68 ± 0.28gh		3.70 ± 0.44de	2.44 ± 0.36ghi		3.49 ± 0.50e	6.60 ± 0.45ab		3.38 ± 0.22ef		2.87 ± 0.85fg
14	0.61 ± 0.55fgh	1.58 ± 0.30c		2.33 ± 0.54b		1.40 ± 0.26cde		0.62 ± 0.40fgh		nd	0.93 ± 0.58defgh		0.90 ± 0.63efgh	1.14 ± 0.28cdef		1.12 ± 0.24cdefg		0.67 ± 0.00fgh
15	2.80 ± 0.60c	4.67 ± 0.47a		1.85 ± 0.32efg		1.49 ± 0.40fgh		0.93 ± 0.35hi		0.48 ± 0.09ij	2.20 ± 0.45cde		3.55 ± 0.27b	2.08 ± 0.65def		1.17 ± 0.33h		1.39 ± 0.14gh
Sum	35.11 ± 1.18d	52.21 ± 1.33b		59.33 ± 2.15a		49.25 ± 1.22c		18.94 ± 1.66j		26.26 ± 0.99hi	25.85 ± 1.15hi		34.81 ± 1.66de	49.04 ± 1.44c		32.62 ± 2.03efg		33.53 ± 1.88def
Polymeric procyanidins	51.73 ± 0.99g	100.47 ± 1.33b		109.67 ± 1.11a		88.37 ± 1.15c		74.33 ± 2.33d		34.60 ± 1.21k	48.46 ± 1.65hi		63.69 ± 1.35f	73.53 ± 0.87d			68.15 ± 0.98e	54.04 ± 2.22g
DP	2.91	3.77		3.98		3.44		4.25		2.43	3.41		3.45	3.24			3.80	3.35
Total	87.73 ± 2.22j	154.71 ± 3.00b		170.38 ± 1.25a		138.97 ± 1.98c		94.06 ± 1.98h		61.45 ± 1.24m	76.37 ± 1.56k		100.03 ± 1.54f	123.75 ± 1.88d			103.81 ± 2.22e	90.87 ± 1.78i
Peak no	***Chaenomeles Japonica***											***Chaenomeles Speciosa***						
	**Cido**		**Red Joy**		**Wild #1**		**Wild #2**		**n1 (New)**			**Nivalis**			**Rubra**			**Simonii**
Phenolic acids																		
4	0.09 ± 0.02f		1.07 ± 0.17cd		0.31 ± 0.15ef		1.15 ± 0.10cd		2.12 ± 0.20b			2.05 ± 0.26b			1.29 ± 0.15cd			0.25 ± 0.05ef
5	0.06 ± 0.01c		0.04 ± 0.01c		0.70 ± 0.22b		nd		0.28 ± 0.16bc			0.19 ± 0.10c			nd			2.80 ± 0.33a
Sum	0.15 ± 0.05g		1.11 ± 0.21def		1.01 ± 0.06def		1.15 ± 0.15def		2.40 ± 0.17b			2.24 ± 0.25b			1.29 ± 0.10de			3.05 ± 0.44a
Flavan-3-ols																		
1	0.38 ± 0.10i		1.88 ± 0.19cde		0.48 ± 0.11hi		1.09 ± 0.17fgh		2.29 ± 0.38abc			2.08 ± 0.30bcd			2.51 ± 0.50ab			0.37 ± 0.14i
2	0.39 ± 0.13cde		0.71 ± 0.19abcde		0.22 ± 0.13e		0.56 ± 0.14abcde		0.86 ± 0.21abc			0.69 ± 0.38abcde			0.99 ± 0.39ab			0.81 ± 0.25abcd
3	nd		1.04 ± 0.24de		nd		0.75 ± 0.28ef		1.20 ± 0.19de			1.37 ± 0.26d			1.40 ± 0.30cd			nd
6	2.23 ± 0.15b		0.84 ± 0.20e		1.17 ± 0.26cde		0.91 ± 0.15de		1.34 ± 0.08cde			1.09 ± 0.33cde			1.13 ± 0.16cde			2.32 ± 0.47b
7	11.40 ± 0.99d		3.39 ± 0.96j		6.19 ± 0.54h		5.36 ± 0.66i		8.40 ± 0.72f			7.15 ± 0.60g			3.80 ± 0.58j			8.49 ± 0.50f
8	1.57 ± 0.50bcd		0.48 ± 0.33g		1.09 ± 0.44defg		0.77 ± 0.20efg		1.57 ± 0.00bcd			1.11 ± 0.24def			0.56 ± 0.10fg			nd
9	4.37 ± 0.22f		1.77 ± 0.11h		4.40 ± 0.43ef		2.31 ± 0.39gh		2.92 ± 0.18g			2.88 ± 0.52g			1.97 ± 0.12h			4.99 ± 0.12de
10	0.36 ± 0.09abc		0.41 ± 0.20abc		0.32 ± 0.15bc		nd		0.53 ± 0.22abc			0.42 ± 0.29abc			0.43 ± 0.08abc			0.83 ± 0.00ab
11	4.05 ± 0.61de		1.54 ± 0.88j		2.61 ± 0.71gh		2.43 ± 0.70hi		3.94 ± 0.59de			3.63 ± 0.20ef			1.75 ± 0.55j			2.79 ± 0.80gh
12	0.49 ± 0.14l		0.39 ± 0.00l		3.21 ± 0.27cde		1.17 ± 0.40k		2.67 ± 0.00efg			4.68 ± 0.33a			1.58 ± 0.22ijk			2.96 ± 0.47ef
13	2.41 ± 0.35ghi		0.89 ± 0.61j		2.16 ± 0.33hi		1.91 ± 0.54i		3.45 ± 0.96ef			2.86 ± 0.46fg			0.82 ± 0.28j			2.55 ± 0.27gh
14	4.74 ± 0.19a		0.87 ± 0.18efgh		0.56 ± 0.11fghi		0.54 ± 0.27ghi		0.72 ± 0.33fgh			1.50 ± 0.21cd			0.38 ± 0.20hi			0.39 ± 0.15hi
15	1.94 ± 0.30efg		0.27 ± 0.08j		2.64 ± 0.33cd		0.45 ± 0.24ij		1.15 ± 0.51h			2.42 ± 0.36cde			0.31 ± 0.36j			1.41 ± 0.22gh
Sum	34.33 ± 1.54de		14.48 ± 1.11k		25.05 ± 2.29i		18.25 ± 1.55j		31.04 ± 1.43g			31.88 ± 2.00fg			17.63 ± 1.55j			27.10 ± 1.48h
Polymeric procyanidins	67.48 ± 1.99e		40.08 ± 1.64j		37.37 ± 1.00jk		51.35 ± 1.70gh		62.31 ± 1.46f			90.83 ± 2.15c			38.92 ± 1.02j			45.83 ± 0.99i
DP	4.18		2.72		3.16		3.87		3.53			3.95			2.70			2.74
Total	101.96 ± 1.14ef		55.67 ± 2.15n		63.43 ± 1.11m		70.75 ± 1.77l		95.75 ± 1.65g			124.95 ± 1.14d			57.84 ± 2.05n			76.79 ± 1.19k

nd, not detected; ± standard deviation; DP, degree of polymerization; value in the same columns followed by different letters are significantly different at *p* ≤ 0.05according to Tukey’s tes.

**Table 4:** Antioxidant (mmol Trolox/100 g dw), α-amylase, α-glucosidase, pancreatic lipase, acetylcholinesterase, butyrylcholinesterase (IC_50_, mg/mL),and 15-lipoxygenase inhibitionactivity (% of inhibition) of various species and cvs. of *Chaenomeles* fruits.

Spcecies	Cultivar	Antioxidant Capacity			In Vitro Inhibition Activities					
		ABTS	FRAP	ORAC	α-amylase	α-glucosidase	Pancreatic Lipase	AChE	BuChE	15-LOX
***Chaenomeles* × *superba***	**Crimson and Gold**	16.03 ± 1.04cdef	16.00 ± 0.99de	54.93 ± 1.11bc	17.49 ± 0.88a	7.03 ± 0.20def	0.29 ± 0.01ab	11.84 ± 0.24efg	10.13 ± 0.97efgh	99.81 ± 0.15a
	**Texas Scarlet**	19.63 ± 0.99ab	17.90 ± 1.22bcde	53.89 ± 1.37cd	14.34 ± 0.48b	5.08 ± 0.22g	0.09 ± 0.00def	17.43 ± 0.56ab	8.90 ± 0.70fghi	43.23 ± 0.73f
	**Nicoline**	20.61 ± 1.13a	21.32 ± 0.85a	51.86 ± 0.90cde	13.88 ± 0.98b	2.67 ± 0.17h	0.07 ± 0.02ef	16.02 ± 0.25bcd	16.14 ± 0.79d	71.24 ± 0.18d
	**Andenken an Karl Ramcke**	18.80 ± 0.88abc	15.51 ± 1.14def	40.38 ± 0.83h	16.28 ± 0.77ab	6.71 ± 0.72defg	0.04 ± 0.02f	13.03 ± 0.81defg	9.96 ± 0.17efgh	74.81 ± 0.18c
	**Pink Lady**	17.65 ± 1.50abcde	17.29 ± 0.63bcde	57.86 ± 1.00b	18.01 ± 0.89a	5.90 ± 0.85fg	0.20 ± 0.10bcd	15.94 ± 0.13bcd	12.14 ± 0.87e	>100.00
	**Colour Trail**	11.02 ± 0.72h	10.56 ± 0.55h	66.59 ± 0.55a	17.56 ± 0.99a	7.95 ± 0.16cde	<0.01	17.53 ± 0.41ab	7.85 ± 0.77hi	75.94 ± 0.29c
	**Flocon Rose**	15.38 ± 1.80defg	15.32 ± 0.88efg	45.25 ± 0.99g	15.59 ± 0.66ab	7.18 ± 0.11def	<0.01	11.73 ± 0.47efg	22.70 ± 0.63bc	98.15 ± 1.00a
	**Hollandia**	18.36 ± 0.63abcd	19.44 ± 1.11abc	40.72 ± 0.78h	16.49 ± 0.32ab	6.10 ± 1.23fg	<0.01	10.73 ± 0.74gh	15.89 ± 0.44d	>100.00
	**Jet Trail**	18.91 ± 0.91abc	20.04 ± 1.37ab	54.80 ± 0.46bc	17.97 ± 1.00a	6.97 ± 0.77def	0.29 ± 0.01ab	12.22 ± 0.65efg	8.09 ± 0.99ghi	>100.00
	**wild**	17.46 ± 0.81bcde	18.45 ± 0.45abcd	50.18 ± 0.89ef	18.25 ± 0.39a	7.17 ± 0.57def	<0.01	11.05 ± 0.57g	31.59 ± 0.95a	>100.00
	**Cameo**	15.24 ± 0.24efg	11.93 ± 0.33h	51.63 ± 1.62de	16.75 ± 0.57ab	8.54 ± 0.34cd	0.12 ± 0.02def	11.56 ± 0.84fg	12.37 ± 1.22e	36.84 ± 0.44g
***Chaenomeles japonica***	**Cido**	18.06 ± 1.52abcde	18.00 ± 0.65bcde	48.48 ± 1.55f	16.47 ± 0.56ab	6.49 ± 0.49efg	0.17 ± 0.00cde	14.75 ± 0.75bcde	16.42 ± 0.31d	42.11 ± 0.56f
	**Red Joy**	13.50 ± 0.50fgh	12.76 ± 1.12fgh	53.43 ± 0.87cd	17.45 ± 0.54a	15.19 ± 0.14a	0.06 ± 0.01ef	7.74 ± 0.34hi	6.06 ± 0.41i	73.31 ± 0.74cd
	**wild #1**	12.41 ± 0.41gh	11.53 ± 0.55h	40.28 ± 0.66h	16.66 ± 0.87ab	6.11 ± 0.19efg	0.35 ± 0.05a	12.14 ± 0.20efg	32.11 ± 1.13a	90.60 ± 0.69b
	**wild #2**	13.72 ± 0.72fgh	12.30 ± 0.62gh	33.99 ± 1.74i	16.11 ± 1.13ab	9.57 ± 0.55c	<0.01	10.13 ± 0.30gh	20.68 ± 0.56c	70.37 ± 0.55d
	**n1 (new)**	16.96 ± 0.96bcde	16.90 ± 0.22cde	51.07 ± 0.77def	16.89 ± 0.98ab	6.09 ± 1.22fg	0.25 ± 0.05abc	14.18 ± 0.49cdef	11.17 ± 0.66efg	66.05 ± 0.99e
***Chaenomeles speciosa***	**Nivalis**	17.54 ± 0.54abcde	16.39 ± 0.47cde	44.30 ± 1.50g	18.48 ± 0.43a	6.56 ± 0.46efg	0.20 ± 0.02bcd	16.52 ± 35bc	11.97 ± 0.20ef	74.81 ± 0.45c
	**Rubra**	10.91 ± 0.91h	10.24 ± 1.20h	33.82 ± 0.49i	18.38 ± 0.77a	5.74 ± 0.84fg	0.04 ± 0.00f	6.65 ± 0.73i	11.07 ± 0.81efg	14.29 ± 0.99i
	**Simonii**	17.37 ± 1.37bcde	17.02 ± 0.98bcde	45.18 ± 1.15g	16.88 ± 1.00ab	12.48 ± 0.68b	0.20 ± 0.05bcd	20.42 ± 0.99a	25.79 ± 0.11b	27.37 ± 0.30h

± standard deviation; value in the same columns followed by different letters are significantly different at *p* ≤ 0.05according to Tukey’s test.

## Supplementary Materials

The following are available online at https://www.mdpi.com/2076-3921/9/1/60/s1, Figure S1: Structural formulas of selected phenolic compounds identified in *Chaenomele*s fruits.
