# Supplementary material for: ABTS On-Line Antioxidant, α-Amylase, α-Glucosidase, Pancreatic Lipase, Acetyl- and Butyrylcholinesterase Inhibition Activity of Chaenomeles Fruits Determined by Polyphenols and other Chemical Compounds
Source: Antioxidants (Basel). 2020 Jan 9;9(1):60. doi: 10.3390/antiox9010060 (PMC7023120; doi:10.3390/antiox9010060)
Supplement: Supplementary file 1 [file antioxidants-09-00060-s001.pdf]

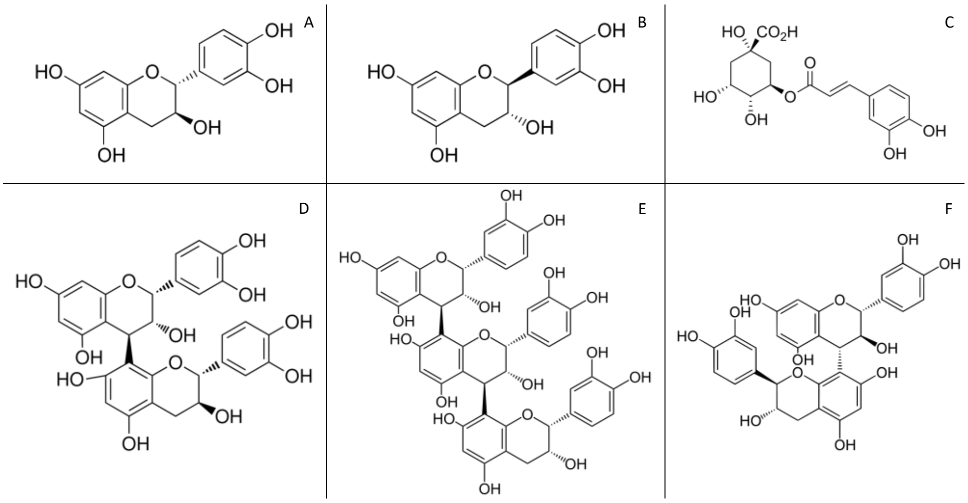

Figure S1. Structural formulas of selected phenolic compounds identified in *Chaenomele*s fruits: A-(+)-Catechin, B-(-)-Epicatechin, C-5-*O*-Caffeoylquinic acid (chlorogenic), D-Procyanidin B2, E-Procyanidin C1, F-Procyanidin B3
